# Association of NAFLD and Insulin Resistance with Non Metastatic Bladder Cancer Patients: A Cross-Sectional Retrospective Study

**DOI:** 10.3390/jcm10020346

**Published:** 2021-01-18

**Authors:** Giovanni Tarantino, Felice Crocetto, Concetta Di Vito, Massimiliano Creta, Raffaele Martino, Savio Domenico Pandolfo, Salvatore Pesce, Luigi Napolitano, Domenico Capone, Ciro Imbimbo

**Affiliations:** 1 Department of Clinical Medicine and Surgery, Federico II Medical School, Via S. Pansini 5, 80131 Naples, Italy; 2 Department of Neuroscience, Reproductive Sciences and Dentistry, University of Naples “Federico II”, Via S. Pansini 5, 80131 Naples, Italy; crocetto@unina.it (F.C.); tina.dvt@tiscali.it (C.D.V.); max.creta@gmail.com (M.C.); raffaele.martino88@yahoo.it (R.M.); pandolfosavio@gmail.com (S.D.P.); salvatorepesce90@gmail.com (S.P.); nluigi89@libero.it (L.N.); ciro.imbimbo@unina.it (C.I.); 3 Clinical Pharmacology Consultant, Via Volturno 27, 80026 Naples, Italy; domenico.capo2016@gmail.com

**Keywords:** bladder cancer, no cancerous bladder diseases, NAFLD, insulin resistance, impaired fasting glucose, prediabetes, type 2 diabetes mellitus

## Abstract

Among risk factors (apart from smoking) likely involved in bladder cancer (BCa), metabolic syndrome (MS), obesity and type 2 diabetes mellitus (T2DM) have been explored with contrasting results. In spite of these studies, there is little data on the association between nonalcoholic fatty liver disease (NAFLD), its main driver, i.e., insulin resistance (IR), and BCa. Implanting a cross-sectional retrospective study we tried to investigate both NAFLD and IR prevalence in a hospital based population of BCa patients. We studied laboratory data from 204 patients with histologically confirmed non metastatic BCa and 50 subjects with no BCa, but with bladder diseases (no Ca BD). We evaluated the presence of NAFLD by the triglycerides/glucose Index (TyG Index), using a cut-off of 0.59 and by the Aspartate Aminotransferase/Alanine Aminotransferase AST/ALT ratio. IR was assessed by the same TyG Index (cut-off 4.68) and the triglycerides/High-Density Lipoprotein HDL ratio (cut-off 2.197). The diagnosis of impaired fasting glucose (IFG), condition of prediabetes, as well as that of T2DM was assessed according to canonical guidelines. The TyG Index predicted NAFLD presence in both groups (*p* = 0.000), but the BCa group showed a major percentage of NAFLD cases with respect to no Ca BD group (59% versus 40%). A greater proportion of IR (47%) in BCa group than in no Ca BD one (37%) was evidenced by the TyG Index with its median value significantly different (*p* = 0.0092). This high rate of IR in the BCa group was confirmed by the triglycerides/HDL ratio (*p* = 0.02). Prediabetes and T2DM were more prevalent in the BCa group than no Ca BD group (*p* = 0.024). In this study a consistent NAFLD presence was found in BCa patients. This is an important comorbidity factor that deserves further consideration in prospective studies. The higher prevalence of NAFLD, IR, prediabetes and T2DM in the BCa group evidences the need that these disorders should be reckoned as adjunct factors that could impact on this cancerous disease.

## 1. Introduction

Bladder cancer (BCa) corresponds to the 4th and 11th most common cancer among men and women, respectively [1]. More than two-thirds of patients present with superficial tumours (stages Ta and T1) that are reckoned as Non Muscle-Invasive Bladder Cancer (NMIBC), [2], while in stage T2a the tumour has spread to the inner half of the muscle of the bladder wall. 

It is important to know about determinants for BCa because there may be modifiable factors that might lower cancer susceptibility and prognosis. Beyond smoking, other risk factors for BCa have been studied, including metabolic syndrome (MS), obesity, and type 2 diabetes mellitus (T2DM) [3,4,5]. Based on large cohorts, T2DM significantly increased the risk of BCa [6,7], but findings from a meta-analysis suggest that individuals with T2DM may have a modestly increased risk of BCa [8] or none according to a population-based case-control study [9]. Still, analysing a vast population, data do not support an association of T2DM with overall bladder cancer incidence from 1992 through 2007 [10]. A disease strictly linked to glucose homeostasis disruption is nonalcoholic fatty live (NAFLD) that recognizes as main driver insulin resistance (IR) [11]. At our best knowledge there is few data on NAFLD and IR in BCa patients.

To evaluate IR, the triglyceride-to-HDL (High-Density Lipoprotein) cholesterol ratio has been proposed as a new index [12]. Recently, studying a middle-aged and elderly population, authors set the optimal threshold value at 2.197, where the corresponding sensitivity and specificity were 72.4 and 65.1% [13]. More stringent cut-offs of triglyceride-to-HDL cholesterol ratio have been proposed by other authors, i.e., >2.5 in women and >3.5 in men [14].

Indeed, another surrogate for identifying IR, compared with euglicemic-hyperinsulinemic clamp, has been recently suggested, labelled triglycerides/glucose index (TyG Index), whose best value for diagnosis of IR has been set to 4.68 mg/dL, with a sensitivity of 96.5% and specificity of 85.0% [15]. The same TyG Index, at cut-offs of 4.58 and 4.59, has been recently adopted for screening two spectra of nonalcoholic liver disease (NAFLD), i.e., fatty liver and nonalcoholic steatohepatitis [16]. Moreover, according to multivariate logistic regression analyses of a clinical investigation, the Aspartate Aminotransferase/Alanine Aminotransferase AST/ALT ratio (normally being <1) has remained an independent predictor of NAFLD [17]. 

Due to scarce data present in literature, we aimed at evaluating in a hospital-based population the prevalence of NAFLD and IR as well as two conditions linked to IR such as prediabetes and T2DM in BCa patients and in subjects with no cancerous bladder diseases/urinary conditions (no Ca BD), evaluating laboratory specific indices.

## 2. Methods

### 2.1. Study Design and Data Source

We retrospectively evaluated the clinical and laboratory data drawn from administrative records of 254 patients admitted to a tertiary University/Hospital for low urinary tract (bladder) diseases undergoing cystoscopy, from January 2017 to December 2019, broken in two groups, i.e., patients with histologically-confirmed, non metastatic BCa *n* = 204 and without BCa, but with bladder diseases (no Ca BD), *n* = 50 (49 chronic cystitis and 1 hemangioma). Eleven patients of the bladder cancer group, and three from the group without bladder cancer were not originally selected due to incomplete data reporting that did not permit a complete statistical analysis.

The paper does not report on primary researching and analysed data were reported on the hospital admission. Our analysis looked at data of these cohorts, respecting complete anonymity and was performed internally as part of an evaluation to improve our quality of care. Patients were diagnosed and treated according to national guidelines and agreements. Testing blood as well as recording all other variables included in our analysis was essential for confirming diagnosis and classifying patients. It was done for each patient without fail and as part of routine care, and was in no way an add-on for purposes of research. For these reasons no ethical approval was requested and informed written consent was not obtained from each subject.

### 2.2. Bladder Cancer Diagnosis

Cystoscopy was followed by Biopsy/Transurethral Resection of Bladder Tumor (TURBT). The stage and the grade were determined based on examining the sample removed during a TURBT according to recent well-accepted guidelines [18].

### 2.3. Metabolic Assessment

Patients were categorised as non-diabetics (normoglycemics) or with prediabetes, diagnosed by impaired fasting glucose (IFG), or suffering from T2DM on the basis of the following criteria, i.e., recently confirmed levels of fasting plasma glucose (FPG) <100 or recently confirmed levels of fasting glucose between 100 and 125 or history of T2DM on antidiabetic treatment as well as recently confirmed levels of fasting glucose >126 mg/dL or higher [19]. IR was appreciated by the means of the TyG Index, calculated with the following formula: Ln (fasting triglycerides (mg/dL) × fasting glucose (mg/dL))/2 using a cut-off of 4.68 and by the triglycerides/HDL ratio using a cut-off of 2.197.

### 2.4. Laboratory Data

As surrogate markers ascertaining the NAFLD presence, the TyG and the AST/ALT ratio were carried out, by setting their cut-offs at 4.59 and >1, respectively. The presence of one drink/day or seven drinks/week for women and 2 drinks/day and 14 drinks/week for men was criterium of exclusion for the diagnosis of NAFLD.

The lipids profile comprehended serum levels of triglycerides, total cholesterol, HDL-cholesterol and LDL-cholesterol. AST, ALT and gamma-GT were analysed as liver enzymes. All these parameters were measured according to in-house procedures. 

### 2.5. Statistics

Data, derived from a normally distributed population, were given as mean plus SD, while for the not normally distributed one, were expressed as median plus 25–75 interquartile range (IQR). For every examined variable the number of observations was pointed out to better interpret results.

Frequency tables were used to assess relationships between categorised variables, studying the Pearson’s chi2. Differences between medians were analysed by the two-sample Wilcoxon rank-sum (Mann-Whitney) test. Kruskal-Wallis equality-of-populations rank test was used to evaluate differences between more than two groups.

As measure of association we chose to study predictions that were carried out by various types of regression techniques. As univariate analysis the linear regression analysis was employed. Being covariate a possible predictive or explanatory variable of the dependent variable, in one model the prediction was adjusted for age.

In suspicion of heteroscedasticity and having detected the presence of few outliers, we analysed the correlation by the robust regression, using Least Absolute Deviations (LAD) Regression. Dealing with a binary dependent variable, the logistic regression the prediction tool was carried out, by which the Odds ratio with related 95% CI was evaluated.

Stata16.1 was the program on which we run statistics (StataCorp LLC 4905 Lakeway Drive College Station, TX 77845-4512, USA).

## 3. Results

### 3.1. Principal Characteristics of the Studied Population

Principal characteristics of the studied population are showed in Table 1.

The gender distribution and the median age showed no difference among BCa and no Ca BD patients, *p* = 0.58, Pearson’s chi2 and *p* = 0.056, two-sample Wilcoxon rank-sum (Mann-Whitney) test, respectively.

The majority of bladder cancers belonged to NMIBCs. Specifically, 182 patients (89%) presented with stages Ta/T1, respectively 112/70, while the patients with stage T2a were only 21. The patients with carcinoma in situ were 13.

**Figure 1:**
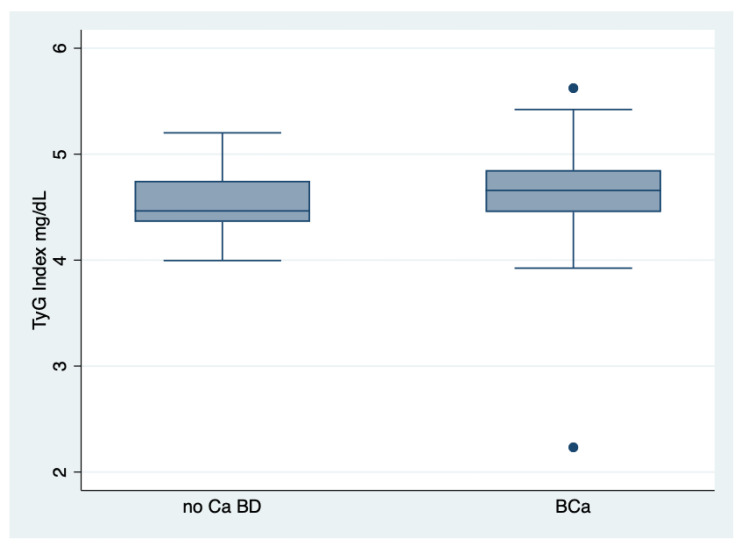
Behaviour of the TyG Index in both groups. TyG Index, triglycerides/glucose index; BCa, bladder cancer; no Ca BD, no cancerous bladder diseases/urinary conditions. There are two outliers among BCa group.

There was no difference in gender concerning the values of TyG index in the whole population, two-sample Wilcoxon rank-sum (Mann-Whitney) test, *p* = 0.330. There was difference between the median value of TyG Index of patients with BCa and that of no Ca BD ones, two-sample Wilcoxon rank-sum (Mann-Whitney) test, *p* = 0.0092, Figure 1.

Frequencies of normal FPG, pre-diabetes, T2DM were different between the two groups.

Pearson chi2 = 8.18, *p* = 0.017. Furthermore, summing up patients with prediabetes and T2DM, there was a major prevalence of them in the BCa (44%) versus no Ca BD group (28%), Pearson chi2 = 7.49, *p* = 0.024, Table 2.

The TyG Index did show a major risk of incident NAFLD (59%) in BCa patients than in no Ca BD patients (40%), by using the best cut-off of 4.59, *p* = 0.06. Interestingly, in the BCa group, the NAFLD percentage (59%) was superior to that obtained summing up the prediabetes and T2DM (44%) rates.

The AST/ALT ratio was slightly increased with respect to the normal value, showing a modest trend in predicting NAFLD, but there was no difference in both groups.

There was a higher percentage of IR in BCa group than no Ca BD group (47 versus 37%, *p* = 0.24) by the TyG Index (cut-off 4.68). At Kruskal-Wallis equality-of-populations rank test, the TyG Index median was different between normoglycemics, prediabetics and T2 DM patients, chi-squared = 8.88 with 2 d.f., *p* = 0.011. BCa patients showed a superior median value of the TyG Index, i.e., 4.66, with respect to that of no CA BD group, i.e., 4.46, *p* = 0.0092. As collateral finding, the median of the TyG Index did not differ through the stages of BCa (out of 159 patients, 77 presented grade 1, none grade 2 and 82 grade 3), two-sample Wilcoxon rank-sum (Mann-Whitney) test, *p* = 0.38.

Interestingly, the triglycerides/HDL ratio (cut-off 2.197) showed a prevalence of 104 patients with IR out of 179 (58%) in BCa group and 19 out 48 (40%) in the group no Ca BD group (*p* = 0.02).

At Kruskal-Wallis equality-of-populations rank test, the median of triglycerides/HDL ratio was also different between normoglycemics, prediabetics and T2 DM patients, chi-squared = 8.880 with 2 d.f., *p* = to 0.011. The value of the triglycerides/HDL ratio was significantly higher, two-sample Wilcoxon rank-sum (Mann-Whitney) test, *p* = 0.022 in BCa patients than in patients without BCa.

### 3.2. Predictions

Age predicted the staging and grading of BCa patients as well as glucose homeostasis categorised as normoglycemia, prediabetes and T2DM in the whole population, Table 3.

The TyG Index did not predict the stages and the grades of BCa patients, but showed a significant prediction of NAFLD presence, as main result, and the glucose homeostasis (presence of prediabetes and T2DM, according to the results of Kruskal-Wallis, see above) in both groups, maintaining its significance when adjusted for age, a noteworthy co-factor, Table 4.

**Figure 2:**
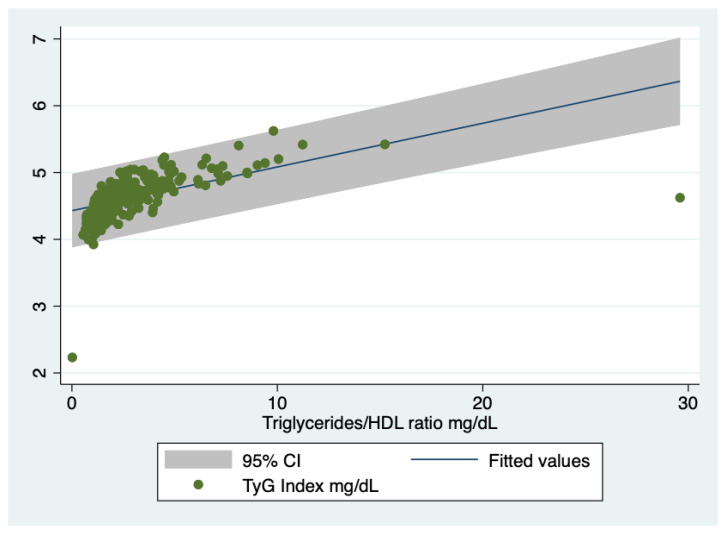
Prediction of the TyG Index by the triglycerides/HDL ratio. TyG Index, triglycerides/glucose index. HDL, high density lipoprotein. There are only two outliers.

Interestingly, the TyG index was predicted by the triglycerides/HDL ratio, i.e., Coef. = 0.06542; Std. Err. = 02806; *t* = 2.33, *p* = 0.021; 95% Conf. Interval. 0.01012–0.12072, Figure 2, even though this index with a cut-off of 3.5 did not predict the glucose homeostasis, i.e., differentiating the three groups (normoglycemics, prediabetics and T2DM patients, *p* = 0.62).

## 4. Discussion

BCa includes diseases that are different for progression and disease-free survival and could be affected by comorbidities. From this perspective, it is useful to explore metabolic risk factors to provide a more satisfactory and individualised therapeutical approach.

The main findings of this retrospective study consist of: the NAFLD presence, evaluated by TyG Index, which was clearly evidenced in both groups but the BCa group showed a major prevalence with respect to the no Ca BD group. Similarly, prediabetes and T2DM were more prevalent in the BCa group than the no Ca BD group. Age played a consistent role in predicting glucose metabolism disorders in both groups as well as grading and staging in the BCa group. IR was more frequent in BCa group than the no Ca BD group, a datum confirmed by both the TyG index and triglycerides/HDL ratio. Interestingly, the median value of the TyG Index of BCa patients, i.e., 4.66 overcame the cut-off of 4.59 mg/dL (index of NAFLD presence), suggesting the appropriateness of choosing this limit. TyG index, well predicting the glucose homeostasis, showed a good reliability.

Regarding the performance of the AST/ALT ratio, it should be stressed that liver enzymes are both insensitive and nonspecific markers of NAFLD [20], accordingly we are not able from these tests to evidence NAFLD presence. In fact, a majority (up to 80%) of subjects with NAFLD have normal serum liver enzyme concentrations [21], even though NAFLD is the most common cause of elevated serum ALT and/or AST [22].

Our results do not confirm those of a recent study showing a lower prevalence of NAFLD (12%) in BCa patients [23]. It should be stressed that data concerning the prevalence of NAFLD of our patients are in keeping with the contextual high prevalence of IR, according to reported values of the TyG Index. Still, the prevalence of NAFLD in no Ca BD patients is in agreement with a recent study showing prevalence rates from NAFLD of 40.3% and 39.2% among 60–74 and  > 74 years old subjects [24]. Interestingly, the percentage of NAFLD in the Bca and no Ca BD patients is superior to that obtained summing up both prediabetes and T2DM rates, even though there was a clear difference in the presence of prediabetes and T2DM comparing the cohorts, lending credence to the fact that IR more than prediabetes and T2DM can somehow determine the NAFLD risk.

What could be the impact of NAFLD comorbidity with its underlying main risk factor, i.e., IR on patients suffering from BCa? IR is characterised by an inappropriate physiologic response in which insensitivity to insulin results in sustained hyperglycaemia and compensatory hyperinsulinemia, even though there is some criticism around this interpretation [25]. Hyperinsulinemia and hyperglycaemia are important regulators of the development of cancer. In fact, insulin signalling via both the PI3K/AKT and MAPK pathways can contribute to cancer cell proliferation [26,27]. What is more, there is a strong correlation between insulin-like growth factor-1 (IGF-1) and NAFLD [28].

IGF-1 signals some of the same pathways as insulin, including PI3K, ERK, AKT, and mTOR, which could increase cancer cell proliferation and impair apoptosis. Furthermore, IGF-1 can increase normal cell cycling, leading to increased risk of mutation and malignant transformation [29].

Last but not least, age was a variable that statistically influenced staging and grading of BCa patients even though we emphasise that in the elderly, age is coincidentally associated with preventable chronic conditions, avoidable exposures, and modifiable risk behaviours that are causally associated with cancer. Commenting on the usefulness of using the TyG index in comparison to another well-known marker of IR (HOMA-IR) we firstly should stress that: (1) TyG Index confronted with its components alone, in a previous very large cohort study, revealed that this parameter exhibits an area under the curve (AUC) of 0.75, higher than that of FPG (0.66) and triglycerides levels (0.71), [30]; (2) TyG Index could be different by race/ethnicity [31]; (3) TyG Index could differ by gender, even though in contrast with previously reported data of literature [15] we did not show that distinction.

Coming back to HOMA-IR, there is debate around its reliability. First of all, HOMA-IR is a relatively extensive method (although there is hardly any consensus on the cut-off points) used in research, but the lack of standardised insulin assays has relatively hindered its development [32]. Anyway, some authors think that the TyG index is not preferable to HOMA-IR [33], while other authors disagree, considering the TyG a measure of interest to identify IR in the general population [34,35,36,37,38,39,40,41,42,43,44].

## 5. Limitations

Firstly, we could have used other surrogates to diagnose NAFLD, but the lack of proposed parameters concerning anthropometric features, evaluating the presence/severity of obesity, did not permit calculating them. Moreover, according to a suggested cut-off of the TyG Index >8 for men and >7.5 for women [45], there was no incidence of NAFLD in our BCa group, but surprisingly neither in the no Ca BD cohort. Regarding the datum of the triglycerides/HDL ratio, evidencing the IR presence according to the cut-off of 2.197, we stress that our findings should be taken with a pinch of salt, because for other authors the limit to confirm IR by the same ratio is 3.5, a value that identified insulin-resistant patients with a sensitivity and specificity comparable to the criteria currently proposed to diagnose the MS [46].

Some discrepancies in categorizing patients as having or not having the disease, based on results of the TyG Index and the triglycerides/HDL ratio, are likely due to arbitrary cut-offs, even though there was a significant relation between the two parameters. Finally, we were not able to carry out a canonical case-control study [47], controls being fewer than cases, although well-defined, i.e., suffering from two different diseases but of the same organ. Finally, due to the study nature we were not able to evaluate a link between these metabolic disorders and the prognosis of BCa.

## 6. Conclusions

In this study the high prevalence of NAFLD, coupled with the high rate of IR in the BCa group, prompts physicians to consider this disorder an important comorbidity that could impact on this cancerous disease. Our research deserves further confirmatory prospective studies.

## 7. Future Directions

Optimal patient management of BCa requires an accurate knowledge of the stage and grade of the disease, beyond comorbidities [48], and an appraisal of the risk of progression and recurrence is required to plan the best course of treatment. Thus, a deep understanding of the metabolic phenotype of BCa will provide novel opportunities for targeted therapeutic strategies [49].

Finally, studying the predictors of recurrence, particular emphasis should be applied to the question whether obtaining detrusor muscle in TURBT of bladder cancer specimens is considered a surrogate marker of resection quality [50].

## Figures and Tables

**Table 1:** Demographic characteristics, comorbidities and studied parameters of the two groups.

Variables	BCa pts	No Ca BD pts	*p*
Age (yrs), median (IQR)	71.5 (64–77)	69 (61–74)	0.056
Gender, F/M (*n* of pts)	34 /170	10/40	0.58
Prediabetes/T2DM (*n* of pts)	48/38 out of 196	10/2 out of 50	0.024
FPG mg/dL, median (IQR)	97.5 (85-121)	91 (81–100)	0.0063
Triglycerides mg/dL, median (IQR)	109 (81–142)	93 (67.5–129.5)	0.083
TyG Index median (IQR)	4.66 (4.45–4.85)	4.46 (4.36–4.74)	0.0092
HDL-Cholesterol mg/dL, median (IQR)	44 (35–53)	48.5 (38.5–55.5)	0.17
Triglycerides/HDL ratio, median (IQR)	2.53 (1.68–3.95)	1.87 (1.33–3.02)	0.022
AST/ALT ratio	1.14 (0.89–1.5)	1.06 (0.85–1.45)	0.27
GGT U/L, median (IQR)	21 (15–34)	24.5 (14–33.5)	0.90
NAFLD *n* **	104 out of 177	19 out of 48	0.06
IR *n* ***	83 out of 177	18 out of 48	0.24

Pts, patients; sec, seconds; F, females; M, males; BCa, bladder cancer; no Ca BD, no cancerous bladder diseases/urinary conditions; IR, insulin resistance; screened *** by the TyG index at a cut-off of 4.68; T2DM type 2 diabetes mellitus; pre-diabetes diagnosed by IFG, impaired FPG, fasting plasma glucose; * evaluated on 203 pts; ** screened by TyG Index selecting the cut-off of steatohepatitis, i.e., 4.59, lightly superior to that of simple steatosis, i.e., 4.58; NAFLD, nonalcoholic fatty liver disease; Ta, noninvasive papillary carcinoma; T1, invades lamina propria; T2a, muscle-invasive disease. TyG, triglycerides/glucose index; AST, aspartate aminotransferase; ALT, alanine aminotransferase; GGT, gamma glutamyl transferase; HDL, high density lipoprotein; IFG, impaired fasting glucose; T2DM, type 2 diabetes mellitus; IQR, interquartile range.

**Table 2:** Frequencies of patients with pre-diabetes, T2DM and normal glycemia.

Diagnosis	Normal FPG	Pre-Diabetes	T2DM	Total
Bca	110	48	38	196
no Ca BD	36	12	2	50
Total	146	60	40	246

Diagnosis: BCa, bladder cancer; no Ca BD, no cancerous bladder diseases/urinary conditions; T2DM, type 2 diabetes mellitus; pre-diabetes diagnosed by IFG, impaired FPG, fasting plasma glucose.

**Table 3:** Prediction of BCa stages and grades and glucose homeostasis by the age of patients.

**Linear Regression, Robust. Number of Observations = 205. R-Squared = 0.051**					
d.v. Staging	Coef.	Std. Err.	*t*	*p* > |t|	95% Conf. Interval
i.v. Age	0.01361	0.00321	4.23	0.000	0.0072–0.01996
**Linear Regression, Robust. Number of Observations = 158. R-Squared = 0.0536**					
d.v. Grading	Coef.	Std. Err.	*t*	*p* > |t|	95% Conf. Interval
i.v. Age	0.02566	0.00756	3.39	0.001	0.0107232–0.0406108
**Linear Regression, Robust. Number of Observations = 248. R-Squared = 0.04**					
d.v. Glucose homeostasis	Coef.	Std. Err.	*t*	*p* >|t|	95% Conf. Interval
i.v. Age	0.01406	0.00339	4.14	0.000	0.00737–0.02074

Staging; Ta, T1 and T2a; Grading, grade 1 and grade 3; glucose homeostasis categorised as subjects with normoglycemia (<100 mg/dL), with IFG/prediabetes (glycemia between 100 and 125 mg/dL), and with glycemia ≥126 mg/dL (T2DM, type 2 diabetes mellitus); d.v., dependent variable; i.v., independent variable. In bold are highlighted the significant ones. The low R-squared, in presence of significance, shows that even noisy, high-variability data (data points fall further from the regression line in graph) can have a significant trend.

**Table 4:** Prediction of NAFLD presence, IFG/T2DM by the triglycerides/glucose index in the whole population and of stages and grades of BCa patients.

**Logistic Regression, Robust. Number of Observations = 232. Pseudo R-Squared = 0.424**					
d.v. NAFLD	Coef.	Std. Err.	*t*	*p* > |t|	95% Conf. Interval
i.v. TyG Index	6.548	1.898	6.48	0.000	3.70–11.560
**Linear Regression, Robust. Number of Observations = 230. R-Squared = 0.094**					
d.v. Glucose homeostasis	Coef.	Std. Err.	*t*	*p* > |t|	95% Conf. Interval
i.v. TyG Index	0.765	0.164	4.66	0.000	0.4422–1.089
**Linear Regression, Robust. Number of Observations = 227. R-Squared = 0.126**					
d.v. Glucose homeostasis	Coef.	Std. Err.	*t*	*p* > |t|	95% Conf. Interval
i.v. TyG Index	0.7276	0.1414	5.14	0.000	0.4487–1.00
Cov. Age	0.0120	0.0036	3.27	0.001	0.004–0.019
**Linear Regression, Robust. Number of Observations = 179. R-Squared = 0.016**					
d.v. Staging	Coef.	Std. Err.	*t*	*p* > |t|	95% Conf. Interval
i.v. TyG Index	−0.2543	0.1319	−1.93	0.056	−0.5148–0.0061
**Linear Regression, Robust. Number of Observations = 140. R-Squared = 0.012**					
d.v Grading	Coef.	Std. Err.	*t*	*p* > |t|	95% Conf. Interval
i.v. Age	−0.3258	0.199	−1.63	0.104	−0.7199–0.0683

Staging: Ta, T1 and T2a; grading, grade 1 grade 2 and grade 3. NAFLD presence evidenced by the TyG index, cut-off 4.59); glucose homeostasis categorised as normoglycemics, <100mg/dL, with pre-diabetes (IFG between 100 and 125 mg/dL), and with T2DM ≥ 126 mg/dL; TyG Index, triglycerides/glucose index; d.v., dependent variable; i.v., independent variable; cov. covariate. In bold are highlighted the significant ones. The low R-squared, in presence of significance, shows that even noisy, high-variability data (data points fall further from the regression line in graph) can have a significant trend.

## Data Availability

The data presented in this study are available on request from the corresponding Author.

## Abbreviations

BCa	Bladder Cancer
NMIBC	Non Muscle-Invasive Bladder Cancer
MS	Metabolic Syndrome
T2DM	type 2 diabetes mellitus
IR	Insulin Resistance
TyG Index	Triglycerides/Glucose Index
NAFLD	NonAlcoholic Fatty Liver Disease
TURBT	Transurethral Resection of Bladder Tumor
IFG	Impaired Fasting Glucose
FPG	Fasting Plasma Glucose
ALP	Alkaline Phosphatase
IQR	Interquartile Range
LAD	Least Absolute Deviations
